# Supplementary material for: Functional analysis of conserved C. elegans bHLH family members uncovers lifespan control by a peptidergic hub neuron
Source: bioRxiv. 2024 Jul 16:2024.07.12.603289. Preprint. [Version 1] doi: 10.1101/2024.07.12.603289 (PMC11275782; doi:10.1101/2024.07.12.603289)
Supplement: 1 — Fig. S1: Molecular phylogeny of the Olig/bHLHb4/b5 subfamily A: Phylogenetic relationship of C. elegans and human Olig/bHLHb4/b5 members. Tree generated at phylogeny.fr (Dereeper et al. 2008) with default parameters. B: Protein sequence alignment of Olig/bHLHb4/b5 members across phylogeny. Created at phylogeny.fr by MUSCLE. Similar residues are colored as the most conserved one (according to BLOSUM62). Colores indicate average BLOSUM62 score: blue 1.5, low 0.5. C: Tabular DiOPT scores of Olig/bHLHb4/b5 members as provided by Marrvel (Wang et al. 2017). One NeuroD homolog is provided as an “outgroup”. D: hlh-32, hlh-17, and hlh-31 are located close to each other in a region of C. elegans chromosome IV that is replete with small RNAs. From the genome browser of WormBase. Fig. S2: hlh-17/31/32null animals show not measurable effects on CEPsh morphology or marker expression A: hlh-17/31/32null animals show no defects in in the expression of the CEPsh marker irIs67 (hlh-17prom::gfp). CEPsh morphology is also unaffected. Representative images of wild type and mutant animals are shown with 10 μm scale bar. Number of animals scored are within each bar. P-values were calculated using Fisher’s exact test. B: hlh-17/31/32null animals show no defects in the expression of glial marker kcc-3(syb4430). Expression in CEPsh is still clearly identifiable. Expression in other glia was also unaffected as indicated by counting the number of kcc-3-expressing cells. Representative images of wild type and mutant animals are shown with 10 μm scale bar. Number of animals scored are shown within each bar. Statistical analysis for CEPsh expression was done using Fisher’s exact test, while that for counting kcc-3-expressing cells was performed using unpaired t-test. Error bars for the scatter plot indicate standard deviation of the mean. Fig. S3: Behavioral analyses of hlh-17/31/32null animals A: hlh-17/31/32null animals do not display defects in the defecation motor program, contrary to a prev [file NIHPP2024.07.12.603289V1-supplement-1.pdf]

bioRxiv preprint doi: https://doi.org/10.1101/2024.07.12.603289; this version posted July 16, 2024. The copyright holder for this preprint (which was not certified by peer review) is the author/funder, who has granted bioRxiv a license to display the preprint in perpetuity. It is made available under aCC-BY 4.0 International license.

A

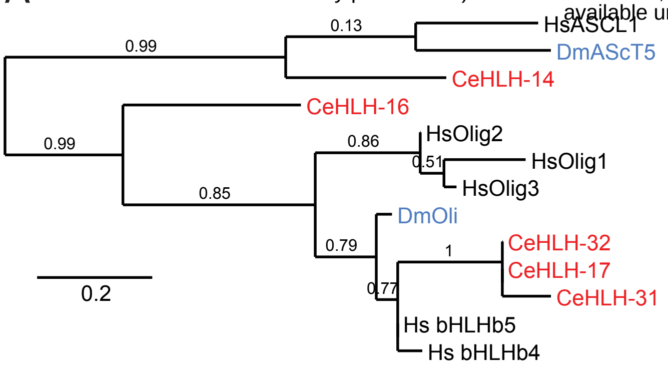

B

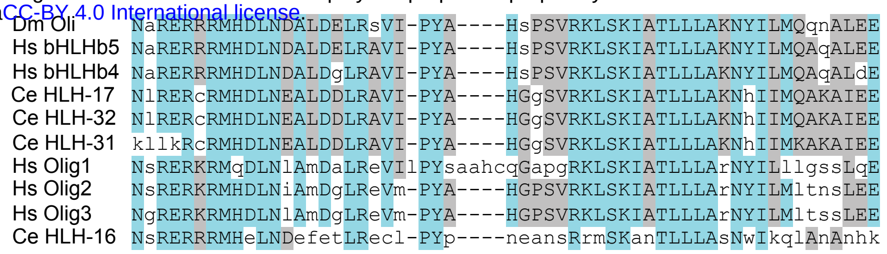

C

	HLH-17	HLH-31	HLH-32	HLH-16	CND-1
Olig1	2/15	2/15	2/15	4/15	1/15
Olig2	3/15	3/15	3/15	2/15	1/15
Olig3	2/15	2/15	2/15	2/15	1/15
bHLHe22	8/15	9/15	9/15	1/15	1/15
bHLHe23	9/15	9/15	10/15	1/15	1/15
NeuroD1	2/15	2/15	2/15	1/15	7/15

D

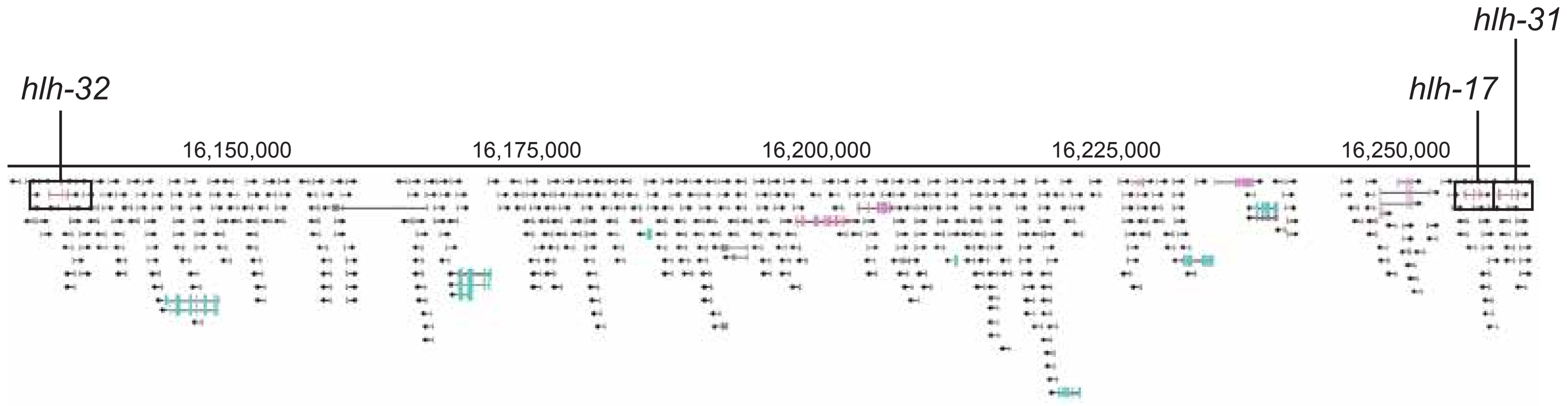

Figure S1

bioRxiv preprint doi: https://doi.org/10.1101/2024.07.12.603289; this version posted July 16, 2024. The copyright holder for this preprint (which was not certified by peer review) is the author/funder, who has granted bioRxiv a license to display the preprint in perpetuity. It is made available under aCC-BY 4.0 International license.

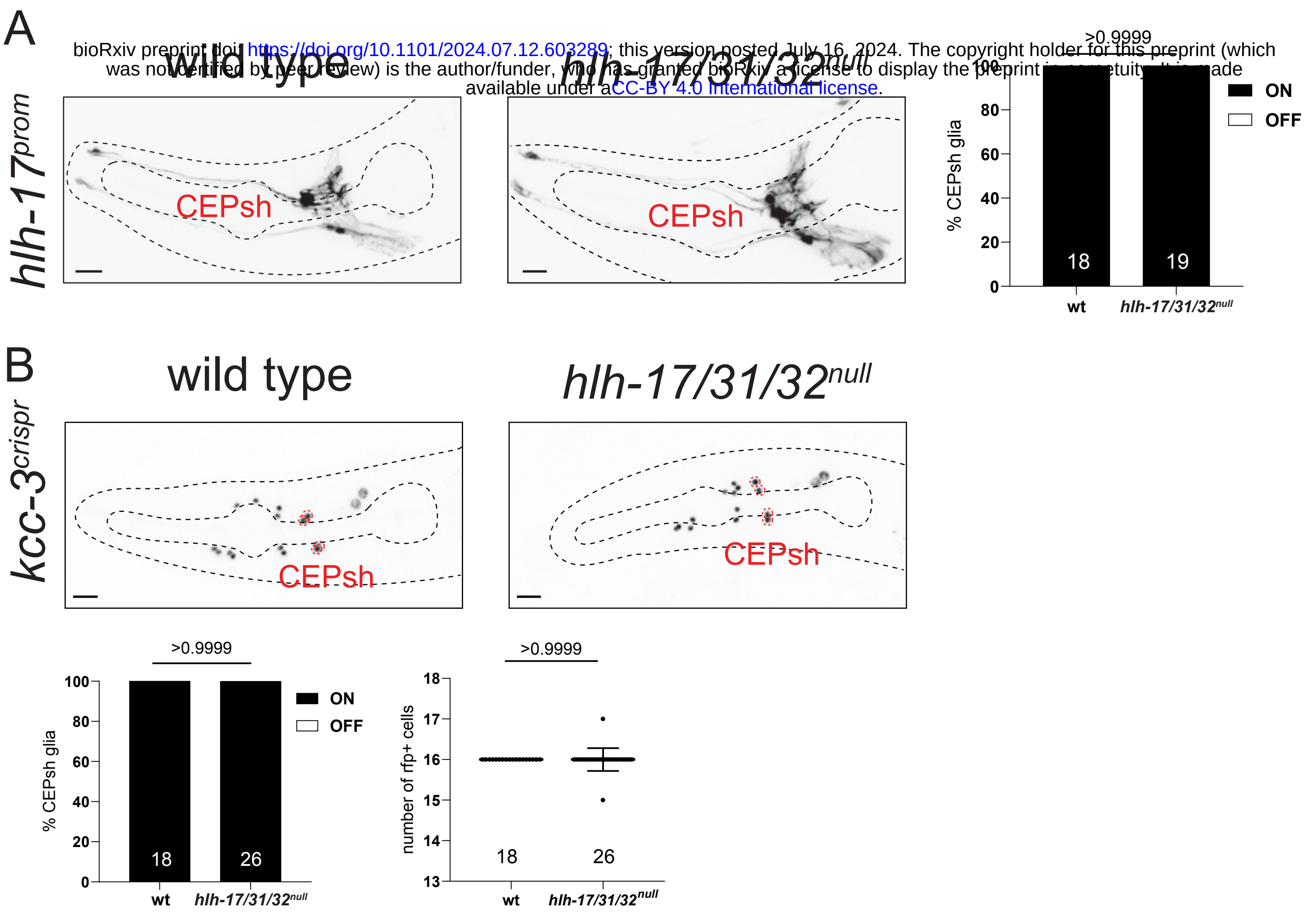

Figure S2

bioRxiv preprint doi: https://doi.org/10.1101/2024.07.12.603289; this version posted July 16, 2024. The copyright holder for this preprint (which was not certified by peer review) is the author/funder, who has granted bioRxiv a license to display the preprint in perpetuity. It is made available under aCC-BY 4.0 International license.

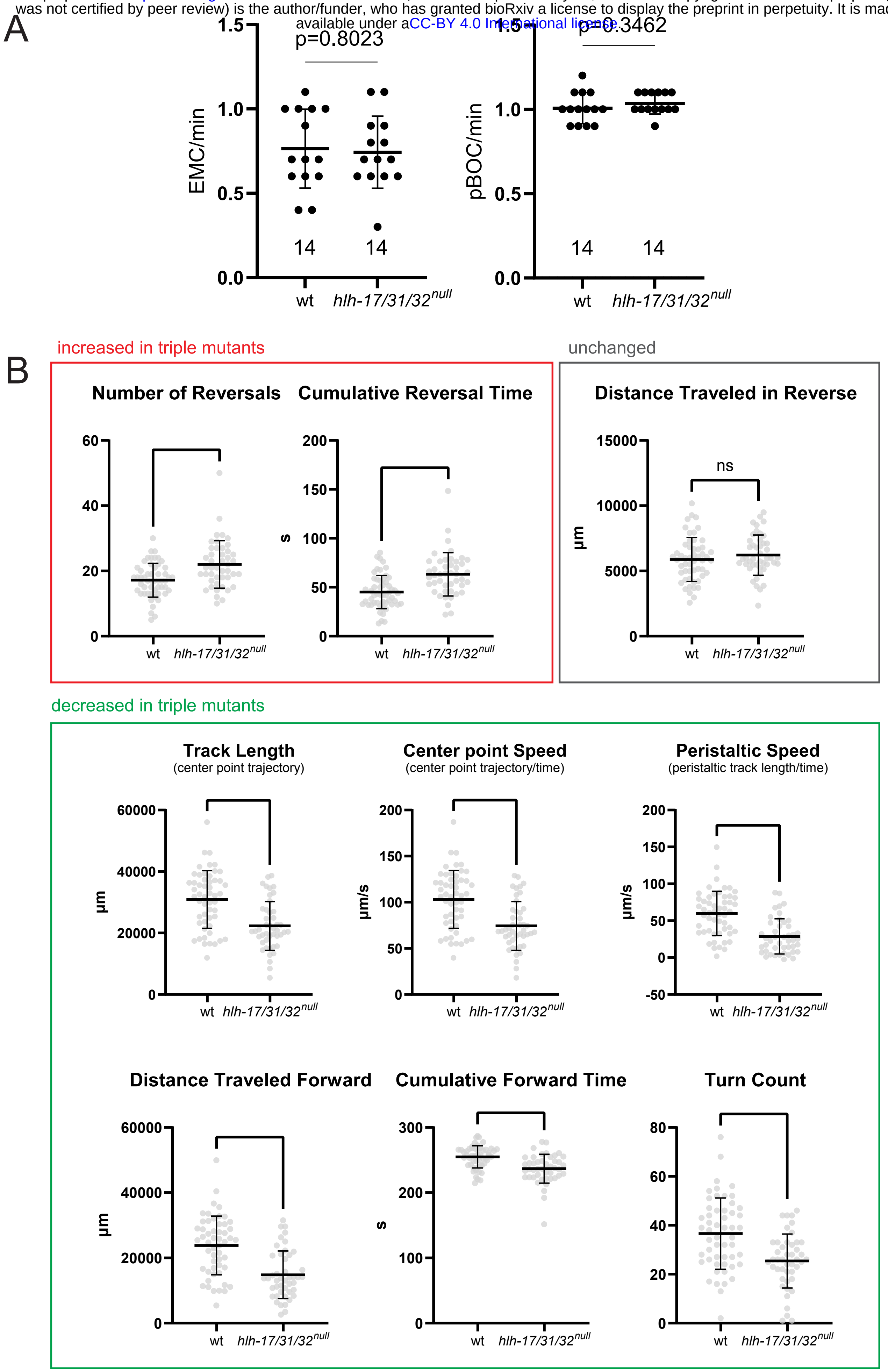

Figure S3

bioRxiv preprint doi: https://doi.org/10.1101/2024.07.12.603289; this version posted July 16, 2024. The copyright holder for this preprint (which was not certified by peer review) is the author/funder, who has granted bioRxiv a license to display the preprint in perpetuity. It is made available under aCC-BY 4.0 International license.

A

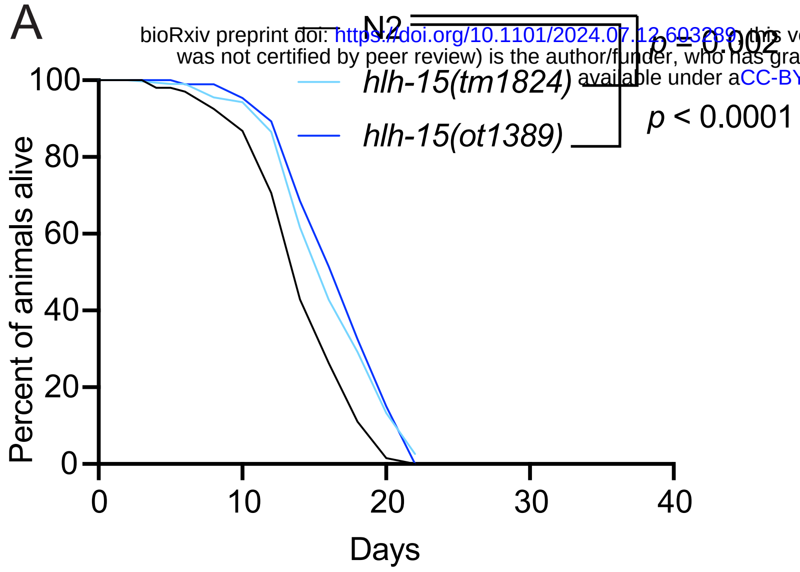

Strain	n	Mean ± SEM (days)	75% dead (days)
N2	105	14.54 ± 0.39	18.00
hlh-15(tm1824)	105	16.71 ± 0.44	20.00
hlh-15(ot1389)	105	17.08 ± 0.41	20.00

B

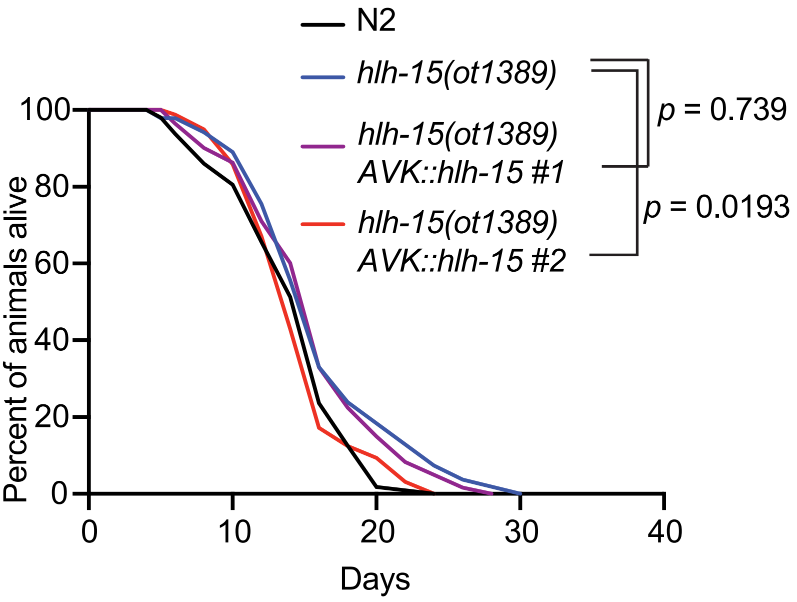

Strain	n	Mean ± SEM (days)	75% dead (days)
N2	105	14.32 ± 0.44	16.00
hlh-15(0t1389)	105	16.23 ± 0.61	18.00
hlh-15(ot1389) AVK::hlh-15 #1	105	15.90 ± 0.56	18.00
hlh-15(ot1389) AVK::hlh-15 #2	105	14.64 ± 0.43	16.00

Figure S4

bioRxiv preprint doi: https://doi.org/10.1101/2024.07.12.603289; this version posted July 16, 2024. The copyright holder for this preprint (which was not certified by peer review) is the author/funder, who has granted bioRxiv a license to display the preprint in perpetuity. It is made available under aCC-BY 4.0 International license.

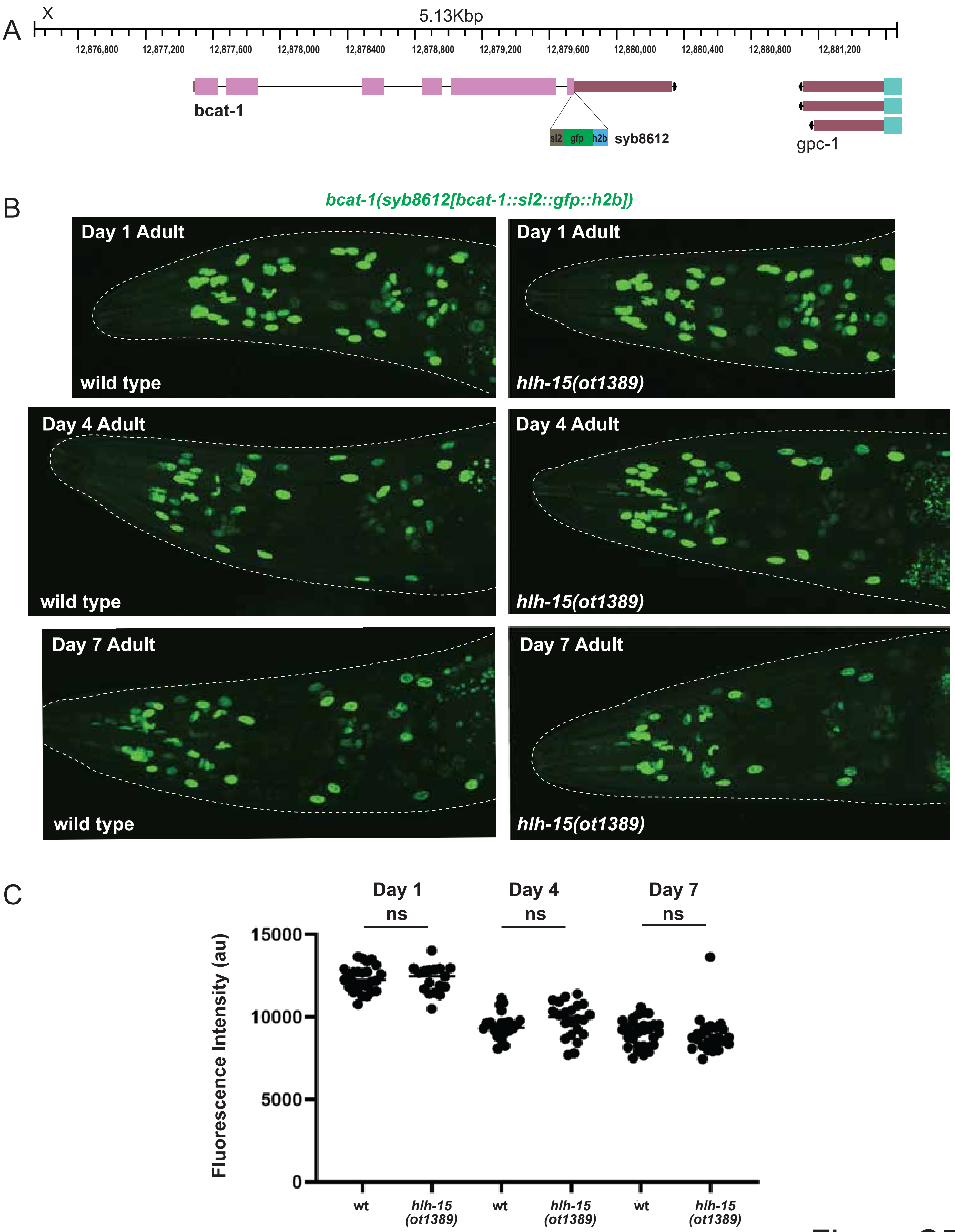

Figure S5

bioRxiv preprint doi: https://doi.org/10.1101/2024.07.12.603289; this version posted July 16, 2024. The copyright holder for this preprint (which was not certified by peer review) is the author/funder, who has granted bioRxiv a license to display the preprint in perpetuity. It is made available under aCC-BY 4.0 International license.

Table S1: Strain list

Strain Name	Genotype	Reference
**Mutants**		
PHX6635	*hlh-17hlh-31(syb6635) IV*	This study
PHX6773	*hlh-17hlh-31(syb6635)hlh-32(syb6773) IV*	This study
OH18840	*hlh-32(ot1347) IV*	This study
OH18614	*hlh-13(ot1388) X*	This study
OH18616	*hlh-13(ot1390) cat-1(syb6486) X; otIs669 V*	This study
OH18615	*hlh-15(ot1389) X*	This study
IU129	*hlh-13(tm2279) X*	NBRP
FX01824	*hlh-15(tm1824) X*	NBRP
MT9455	*tbh-1(n3247) X.*	PMID: 15848803
MT13113	*tdc-1(n3419) II.*	PMID: 15848803
**Transgenes**		
OH15363	*otIs669 (NeuroPAL) him-5(e1490) V*	PMID: 33378642
NY2078	*ynIs78[flp-8p::gfp] X*	PMID: 15236235
OH19054	*pha-1 (e2123) III; otEx8199 [sshk-1p::gfp, pha-1(+)]*	This study
VPR839	*unc-119(ed4) III; irIs67[hlh-17p::gfp + unc-119(+)]*	PMID: 24312354
OH19264	*hlh-15(ot1389) X; otEx8247[flp-1p::ghlh-15::SL2::tagRFP, ttx-3p::gfp]*	This study
OH19265	*hlh-15(ot1389) X; otEx8248[flp-1p::ghlh-15::SL2::tagRFP, ttx-3p::gfp]*	This study
ZX966	*zxIs28[pflp-1(trc)::ICE; pmyo-2::mCherry]*	PMID: 30392795
VL12	*unc-119(ed3) III; wwEx42[hlh-15p::GFP, unc-119(+)]*	PMID: 19632181
OH2007	*nIs107[tbh-1::gfp, lin-15(+] III*	PMID: 15848803
MU1085	*bwIs2[flp-1::GFP, rol-6(su1006)]*	PMID: 10648229
LX811	*vsIs33[dop-3::RFP] V; lin-15B&lin-15A(n765) X*	PMID: 15378064
OP707	*unc-119(tm4063) III; wgIs707[sptf-1::TY1::EGFP::3xFLAG + unc-119(+)].*	PMID: 16990816
CX18236	*egl-3 (nu1711) V; KyEx6532 [flp-1p(513 bp)::CRE (20 ng/uL) + elt-2p::nls::GF*	PMID: 38573858
**Reporter alleles**		
PHX6303	*hlh-17(syb6303[hlh-17::gfp]) IV*	This study
PHX6112	*hlh-31(syb6112[hlh-31::gfp]) IV*	This study
PHX6078	*hlh-32(syb6078[hlh-32::gfp]) IV*	This study
PHX7685	*hlh-13(syb7685 [hlh-13::gfp]) X*	This study
PHX7688	*hlh-15(syb7688 [hlh-15::gfp]) X*	This study
PHX4430	*kcc-3(syb4430[kcc-3::sl2::TagRFP-T::h2b]) II*	This study
PHX3320	*nlp-49(syb3320[nlp-49::T2A::3XNLS::gfp]) X*	This study
PHX8612	*bcat-1(syb8612[bcat-1::SL2::GFP::H2B]) X*	This study
OH18694	*col-105(syb6767[col-105::sl2::gfp::h2b]) him-8(e1489) IV*	This study
OH19033	*pdf-1(syb3330[pdf-1::T2A::3XNLS::GFP]) III; otIs669 V him-5(e1490) V.*	This study
OH18061	*flp-7(syb5413[flp-7:sl2:GFP::H2B]) X; otIs669 him-5(e1490) V*	This study
MCP385	*twk-47(bab385[twk-47::wrmScarlet]) I*	Gift from T. Boulin lab
PHX4374	*flp-32(syb4374[flp-32:SL2:GFP::H2B]) X.*	PMID: 37935195
PHX6148	*nlp-50(syb6148[nlp-50::sl2::gfp::h2b]) II*	PMID: 34759317
PHX4512	*nlp-69(syb4512[nlp-69::sl2::gfp::h2b]) V*	PMID: 36067313
PHX7768	*tdc-1(syb7768[tdc-1::sl2::gfp::h2b]) II*	PMID: 38895397
PHX6486	*cat-1(syb6486[cat-1::SL2::gfp::H2B]) X.*	PMID: 38895397
PHX4491	*unc-17(syb4491[unc-17::T2A::GFP:H2B]) IV*	PMID: 35324425
PHX4595	*tkr-1(syb4595 [tkr-1::SL2::GFP::H2B]) III*	PMID: 37935195
OH18107	*unc-42(ot986) [unc-42::GFP] V; him-8(e1489) IV*	PMID: 34165428
OH16380	*nlp-45(ot1032[nlp-45::T2A::GFP::H2B]) X*	PMID: 34759317
OH14070	*bnc-1(ot845[bnc-1::mNeonGreen::AID]) V.*	PMID: 28056346
